
JOURNAL INFORMATION
==============================
NLM Title Abbreviation: Wirtschaftsdienst
Journal ID: Wirtschaftsdienst
NLM Journal ID: 101086612

Wirtschaftsdienst (Hamburg, Germany : 1949)

ISSN: 0043-6275

ARTICLE INFORMATION
==============================
PMCID: PMC7441475
PMID: 32843775
DOI: 10.1007/s10273-020-2724-1
Article ID: 2724
Article version: 1
Subjects: Ökonomische Trends

Subventionsschub durch Corona?

Subsidy boost from Corona?

Laaser Claus-Friedrich claus-friedrich.laaser@ifw-kiel.de
1
Rosenschon Astrid Astrid.Rosenschon@ifw-kiel.de
2
Schrader Klaus klaus.schrader@ifw-kiel.de
3
1 grid.462465.7 0000 0004 0493 2817 Zentrum Wirtschaftspolitik, Public Relations Zentrum, Institut für Weltwirtschaft, Kiellinie 66, 24105 Kiel, Deutschland
2 grid.462465.7 0000 0004 0493 2817 Prognosezentrum, Institut für Weltwirtschaft, Kiellinie 66, 24105 Kiel, Deutschland
3 grid.462465.7 0000 0004 0493 2817 Schwerpunktanalysen, Institut für Weltwirtschaft, Kiellinie 66, 24105 Kiel, Deutschland
Electronic publication date: 2020 Aug 21
Print publication date: 2020
Volume: 100
Issue: 8
First page: 640
Last page: 642
Copyright: © Der/die Autor(en) 2020
License: Open Access Dieser Artikel wird unter der Creative Commons Namensnennung 4.0 International Lizenz veröffentlicht, welche die Nutzung, Vervielfältigung, Bearbeitung, Verbreitung und Wiedergabe in jeglichem Medium und Format erlaubt, sofern Sie den/die ursprünglichen Autor(en) und die Quelle ordnungsgemäß nennen, einen Link zur Creative Commons Lizenz beifügen und angeben, ob Änderungen vorgenommen wurden.
Die in diesem Artikel enthaltenen Bilder und sonstiges Drittmaterial unterliegen ebenfalls der genannten Creative Commons Lizenz, sofern sich aus der Abbildungslegende nichts anderes ergibt. Sofern das betreffende Material nicht unter der genannten Creative Commons Lizenz steht und die betreffende Handlung nicht nach gesetzlichen Vorschriften erlaubt ist, ist für die oben aufgeführten Weiterverwendungen des Materials die Einwilligung des jeweiligen Rechteinhabers einzuholen.
Weitere Details zur Lizenz entnehmen Sie bitte der Lizenzinformation auf http://creativecommons.org/licenses/by/4.0/deed.de.
License URL: https://creativecommons.org/licenses/by/4.0/

issue-copyright-statement: © The Author(s) 2020

==============================

Am 3. Juni 2020 hat die Große Koalition ein Maßnahmenbündel mit einem Volumen von rund 170 Mrd. Euro geschnürt, das die Corona-Folgen bekämpfen, den Wohlstand sichern und die Zukunftsfähigkeit stärken soll (BMF, 2020; Boysen-Hogrefe, 2020). Dabei handelt es sich um ein Konjunktur- und Krisenbewältigungspaket, das als kurzfristige Krisenhilfe Konjunkturimpulse setzen soll. Darüber hinaus soll durch ein Zukunftspaket die Wirtschaft langfristig durch Investitionen in Spitzentechnologien gefördert werden. Entsprechend enthält dieses kombinierte Corona-Programm Maßnahmen, die zumindest partiell den erlittenen wirtschaftlichen Schaden durch die Pandemiebekämpfung ausgleichen und Corona-bedingte Existenzbedrohungen abwehren sollen. Insofern leistet der Staat denjenigen eine Art Schadenersatz, die ohne Krise und Infektionsschutzmaßnahmen nicht in wirtschaftliche Bedrängnis gekommen wären. Darüber hinaus sind auch Maßnahmen vorgesehen, die den Charakter von Subventionen haben. Schon vor dem Corona-Programm hatten Bund und Länder Hilfsprogramme in beträchtlichem Umfang aufgelegt. Die Einzelposten zeigen kaum einen Subventionscharakter, sondern stellen Schadenersatz für Aktivitätsverbote oder andere Kategorien der Staatsausgaben wie Sozial- oder Forschungsausgaben dar (Laaser und Rosenschon, 2020).

Die Einordnung der Corona-Subventionen erfolgt nach den Bewertungskriterien der Kieler Subventionsampel. Dabei werden die Maßnahmen mit Subventionscharakter nach den Ampelsignalen rot, gelb-orange, gelb oder grün gegliedert (Laaser und Rosenschon, 2018; 2019). Mit der Ampelfarbe ist jeweils ein unterschiedliches Maß an vermuteter Schädlichkeit der einzelnen Maßnahmen verbunden, sodass durch die Größe der Aggregate auch Aussagen über die Allokationswirkungen insgesamt getroffen werden können. Daneben enthält das Corona-Programm Elemente, bei denen sich Maßnahmen mit Subventionscharakter mit eher infrastrukturellen oder allgemeinen Staatsausgaben mischen. Sie werden hier als graue Kategorie bezeichnet. Schließlich wird die temporäre Mehrwertsteuersenkung dokumentiert, weil sie per se Auswirkungen auf die Steuervergünstigungen hat. In Tabelle 1 sind die Positionen des Corona-Programms dargestellt, bei denen a priori ein Subventionscharakter vermutet werden kann. Insgesamt ergibt die Analyse, dass 101,5 Mrd. Euro (60,7 % des Pakets) potenziell subventionsrelevant sind. Die temporäre Absenkung der Mehrwertsteuersätze (A1) wird als formaler Aspekt dokumentiert und erhält kein Ampelsignal. Diese Maßnahme beeinflusst insofern die Höhe der in Deutschland vergebenen Subventionen, als sich damit die auf die Mehrwertsteuer gerichteten Steuervergünstigungen vom Betrag her verringern, soweit die Privilegien selbst unverändert bleiben.

Ein rotes Ampelsignal erhalten fragwürdig erscheinende Positionen, die aus gesamtwirtschaftlicher Sicht entfallen sollten. Das gilt etwa für die Maßnahmen zum Austausch von Fahrzeugflotten zugunsten von Elektrofahrzeugen (B35b, B35d und B35e): Im Rahmen des Postens B35b wird unter anderem die Kaufprämie für Elektroautos auf 6.000 Euro erhöht. Die einseitige Förderung der Elektromobilität ist kritisch zu sehen, da staatlicherseits eine bestimmte Technologie bevorzugt wird, obwohl nicht feststeht, ob es sich hierbei um den Königsweg für eine nachhaltigere Mobilität handelt. Das Bonus-Programm für Zukunftsinvestitionen der Fahrzeughersteller und der Zulieferindustrie (B35c) ist eine spezifische Zuwendung an die Automobilindustrie und ihre Zulieferer.

Der Zuschuss aus Haushaltsmitteln zur Senkung der EEG-Umlage (A3) ist ein Fortschritt, weil eine Preissubvention von den Energieverbrauchern an die Produzenten erneuerbarer Energien zum Teil in eine haushaltsrelevante Finanzhilfe verwandelt wird. Es bedarf jedoch einer grundlegenden Reform der Energiepolitik, durch eine Absenkung der Stromsteuer, eine $CO_2$-Bepreisung, eine Einbeziehung aller Sektoren in das europäische $CO_2$-Lizenzsystem und eine technologieneutrale Förderung der Forschung und Entwicklung im Energiesektor (Felbermayr, 2020). Aufgrund eines bislang fehlenden $CO_2$-Preises sind die Finanzhilfen für den Erhalt der Wälder (A17), für die Umstellung der Flugzeugflotten auf Maschinen neuester Bauart mit geringeren Emissionen (B35l) und für das $CO_2$-Gebäudesanierungsprogramm (B39) widersprüchlich. Problematisch ist nicht das Ziel der Klimaschutzmaßnahmen, sondern deren Ausgestaltung.

Ein gelbes Ampelsignal erhalten Subventionen, wenn der gesamtwirtschaftliche Nutzen zwar begründbar, aber

Tabelle 1

**Subventionsrelevanz der Maßnahmen des Corona-Programms vom 3. Juni 2020**

| Maßnahmenkategorie (mit Nr. in BMF 2020) | Finanzbedarf Mio. Euro | Anteil am Gesamtprogramm[a] in % | Ampelfarbe, Kieler Subventionsampel |
|---|---|---|---|
| (I) Temporäre Mehrwertsteuersenkung (A1) | 20.000[b] | 12,0[b] | --[b] |
| (II) Finanzhilfen/Steuervergünstigungen mit rotem Ampelsignal | | | |
| Austausch von Fahrzeugflotten zugunsten von Elektrofahrzeugen (B35b) | 2.200 | | rot[c] |
| Bonus-Programm für Zukunftsinvestitionen der Fahrzeughersteller und der Zulieferindustrie (B35c) | 2.000 | | rot |
| Flottenaustauschprogramm „Sozial und Mobil" (B35d) | 200 | | rot[c] |
| Flottenaustauschprogramm Handwerker und KMU (B35e) | 0[d] | | rot[c] |
| Zwischensumme rot | 4.400 | 2,6 | |
| (III) Finanzhilfen/Steuervergünstigungen mit gelb-orangem Ampelsignal | | | |
| Zuschuss aus Haushaltsmitteln zur Senkung der EEG-Umlage (A3) | 11.000 | | gelb-orange |
| Finanzhilfe für den Erhalt und die nachhaltige Bewirtschaftung der Wälder (A17) | 700 | | gelb-orange |
| Umstellung der Flugzeugflotten auf Maschinen neuester Bauart mit geringeren Emissionen (B35l) | 1.000 | | gelb-orange |
| $CO_2$-Gebäudesanierungsprogramm (B39) | 2.000 | | gelb-orange |
| Zwischensumme gelb-orange | 14.700 | 8,8 | |
| (IV) Finanzhilfen/Steuervergünstigungen mit gelbem Ampelsignal | | | |
| EU-Notifizierung von Länderhilfen für Fahrgeldausfälle von ÖPNV-Unternehmen (A21) | 0[e] | | gelb |
| Einmalige Erhöhung der Regionalisierungsmittel für den SPNV (A22) | 2.500 | | gelb[f] |
| Finanzhilfen für Sportstätten (A23) | 150 | | gelb |
| Aufstockung der Gemeinschaftsaufgabe GRW-Finanzhilfen (A31) | 500 | | gelb |
| Reduktion der Mitfinanzierungspflichten bei anwendungsorientierter Forschung (B33) | 1.000 | | |
| Förderung der projektbezogenen Forschung (B34) | 300 | | |
| Förderung der Ladezellen-Infrastruktur, Elektromobilität-F&E und Batteriezellforschung (B35f) | 2.500 | | gelb |
| Förderung des Austausches der Mobilfunk-Zugendgeräte (B35h) | 150 | | gelb |
| Aufstockung der Förderung für E-Busse im Stadtverkehr und deren Ladeinfrastruktur (B35i) | 1.200 | | |
| Innovationsförderung im Bereich der Schifffahrt (B35k) | 1.000 | | gelb |
| Vorlage einer „Nationalen Wasserstoffstrategie" (B36) | 7.000 | | gelb[g] |
| Etablierung außenwirtschaftlicher Partnerschaften zur Wasserstoffproduktion (B37) | 2.000 | | gelb[g] |
| Förderung der Innovationskraft der Hersteller von Kommunikationstechnologien 5G und 6G (B45) | 2.000 | | gelb |
| Entbürokratisierung der Förderung des Glasfaser-Breitbandausbaus und Aufbau einer Mobilfunkinfrastrukturgesellschaft des Bundes zum Ausbau eines flächendeckenden 5G-Netzes (B46) | 0[d] | | gelb |
| Inländische Produktion wichtiger Arzneimittel und Medizinprodukte (B52) | 1.000 | | gelb |
| Zwischensumme gelb | 21.300 | 12,7 | |
| (V) Finanzhilfen/Steuervergünstigungen mit grünem Ampelsignal | | | |
| Programm für Überbrückungshilfen zur Existenzsicherung von KMU (A13) | 25.000[h] | | grün |
| Maßnahmen zur Stabilisierung gemeinnütziger Organisationen (A15) | 900 | | grün |
| Coronahilfen im Kulturbereich (A16) | 1.000 | | grün |
| KMU-Prämien für Ausbildungsplatzangebote (A30) | 500 | | grün |
| Beschleunigte Abschreibung von digitalen Wirtschaftsgütern, Förderung digitaler Infrastruktur und beschleunigter digitaler Transformation von KMU (B42) | 1.000 | | grün |
| Zukunftsprogramm Krankenhäuser (B51) | 3.000 | | grün |
| Förderung der Entwicklung von Impfstoffen (B53) | 750 | | grün |
| Zwischensumme grün | 32.150 | 19,2 | |
| (VI) Duale Maßnahmen, sowohl aus allgemeinen Staatsausgaben als auch aus Subventionen bestehend (grau) | | | |
| Zusätzliche Erhöhung des Eigenkapitals der Deutschen Bahn AG (B35g) | 5.000 | | grau |
| Förderung der Künstlichen Intelligenz (B43) | 2.000 | | grau |
| Förderung der Quantentechnologie (B44) | 2.000 | | grau |
| Zwischensumme grau | 9.000 | 5,4 | |
| Gesamtsumme subventionsrelevante Maßnahmen aus dem Corona-Paket | 101.550 | 60,7 | |
| Davon subventionserhöhende Beträge[i] | 72.550 | 43,4 | |
| Nachrichtlich: Summe des Gesamtprogramms laut BMF (2020) | 167.240 | 100,0 | |

[a] In % des Gesamtprogramms einschließlich nicht subventionsrelevanter Posten; in Klammern die Nummerierung der Positionen im Konjunktur- und Krisenbewältigungspaket (A) und Zukunftspaket (B). [b] Die Maßnahme senkt zwar auch unveränderte Mehrwertsteuer-bezogene Steuervergünstigungen und müsste teilweise ein negatives Vorzeichen erhalten, ist aber im Programm als Finanzbedarf der Gesamtmaßnahme einschließlich der Mindereinnahmen wegen der gesenkten Steuersätze selbst gekennzeichnet. [c] Sofern die Technologieförderung technologieoffener wird, Verlagerung in die gelbe Kategorie möglich. [d] Keine zusätzlichen Mittel vorgesehen. [e] Nur verwaltungstechnische Unterstützung. [f] Mit Tendenz zu hellgrün. [g] Bei alleiniger Förderung dieser Technologie in die rote Kategorie einzuordnen. [h] Die Summe von 25 Mrd. Euro soll aus nicht ausgeschöpften bestehenden Programmen finanziert werden. [i] Ohne temporäre Mehrwertsteuersenkung (I) und ohne graue Posten (VI).

Quelle: BMF (2020); eigene Zusammenstellung und Berechnungen.

umstritten ist und Kürzungspotenziale zu vermuten sind. Länderhilfen für Fahrgeldausfälle im ÖPNV (A21) und die einmalige Erhöhung der Regionalisierungsmittel für den SPNV (A22) sind Finanzhilfen, die prinzipiell sinnvoll sind, aber einer Kostenkontrolle bedürfen. Die Finanzhilfen für Sportstätten (A23) wären für den Breitensport relativ unschädlich, für den Profisport dagegen zweifelhaft. Die Aufstockung der Gemeinschaftsaufgabe GRW (A31) ist eine klassische regionalpolitische Subvention. Bei der Reduktion der Mitfinanzierungspflichten bei anwendungsorientierter Forschung (B33) sowie der Förderung der projektbezogenen Forschung (B34), des Austauschs der Mobilfunk-Zugendgeräte bei Eisenbahnverkehrsunternehmen (B35h), von Innovationen im Bereich der Schifffahrt (B35k), der Innovationskraft der Hersteller von Kommunikationstechnologien 5G und 6G (B45) sind Subventionen, bei denen die Förderung über die originäre staatliche Rolle, Grundlagenforschung zu fördern, hinausgeht. Bedenken gegen die einseitige Förderung von Elektroautos gelten grundsätzlich auch für die Förderung der Ladezellen-Infrastruktur, Elektromobilität-F&E und Batteriezellforschung (B35f) sowie der E-Busse im Stadtverkehr und deren Ladeinfrastruktur (B35i). Hier mag eine Anschubfinanzierung eher gerechtfertigt sein, um das Problem bei einer flächendeckenden Versorgung der Fahrzeuge mit Energie zu entschärfen. Für sich genommen wären eine Nationale Wasserstoffstrategie (B36) und die Etablierung außenwirtschaftlicher Partnerschaften zur Wasserstoffproduktion (B37) eine auf bestimmte Technologie zugeschnittene Subvention, allerdings wird damit die bisherige Einseitigkeit der Förderung der Elektromobilität abgeschwächt und technologieoffener gestaltet. Die Entbürokratisierung der Förderung des Glasfaser-Breitbandausbaus und Aufbau einer Mobilfunkinfrastrukturgesellschaft des Bundes zum Ausbau eines flächendeckenden 5G-Netzes (B46) hat teilweise Infrastrukturcharakter, aber die Arbeitsteilung zwischen privatem Lizennehmer und Staat scheint nicht effizient zu sein. Die Inländische Produktion wichtiger Arzneimittel und Medizinprodukte (B52) ist im Unterschied zu anderen Maßnahmen im Medizinbereich skeptisch zu beurteilen. Sowohl für die staatliche Vorsorge als auch für die normale Verfügbarkeit kommt es darauf an, dass diversifizierte Lieferketten und verlässliche vertragliche Bindungen bestehen, um auf die notwendigen Produkte zugreifen zu können.

Ein grünes Ampelsignal erhalten Subventionen, bei denen ein Abbau nicht infrage kommt, etwa weil sie unabweisbar sind. Die Programme für Überbrückungshilfen zur Existenzsicherung von KMU (A13), gemeinnützigen Organisationen (A15) und im Kulturbereich (A16) sind als Schadenersatz für untersagte wirtschaftliche Aktivitäten anzusehen. Bei den KMU-Prämien für Ausbildungsplatzangebote (A30) erscheinen die mit ihnen einhergehenden Verzerrungen durch die Nähe zur Bildungspolitik wenig schädlich. Auch die Förderung digitaler Infrastruktur und Transformation von KMU (B42) erscheint wenig problematisch, da ein die ganze Wirtschaft umfassender Strukturwandel unterstützt werden soll. Beim Zukunftsprogramm Krankenhäuser (B51) zeigt die aktuelle Corona-Pandemie, dass das Vorhalten von Reservekapazitäten essenziell ist. Zumindest die Förderung von Notfall-Kapazitäten ist angesichts unbekannter Pandemien als staatliche Vorsorgeaufgabe anzusehen. Auch die Förderung der Entwicklung von Impfstoffen (B53) erscheint als seuchenvorbeugende Vorsorgemaßnahme vertretbar, obwohl die Entwicklung von COVID-19-Impfstoffen erhebliche private Erträge versprechen dürfte.

Grundsätzlich sind Eigenkapitalerhöhungen durch den Eigentümer wie bei der Deutschen Bahn (B35g) nicht subventionsrelevant. Der Erhöhungsbeitrag dient zwar zum Verlustausgleich, jedoch werden hier vorwiegend Verluste aufgrund des spezifischen Verkehrsrückgangs infolge des Lockdowns ausgeglichen. Selbst wenn sich bei der Förderung der Künstlichen Intelligenz (B43) und der Quantentechnologie (B44) Ansatzpunkte für Anwendungsforschung ergeben, handelt es sich doch auch um Maßnahmen, die näher bei der staatlich zu fördernden Grundlagenforschung angesiedelt sind.

Die hier vorgenommene vorläufige Auswertung des kombinierten „Corona-Programms“ macht deutlich, dass eine ganze Reihe von Hilfsmaßnahmen zum Teil oder gänzlich subventionsrelevant ist. Es muss sich nun zeigen, ob es sich um einen einmaligen krisenbedingten Niveausprung oder einer Verstetigung bei den Subventionen handelt.

Dr. Claus-Friedrich Laaser und Dr. Astrid Rosenschon sind Mitarbeiter des Instituts für Weltwirtschaft in Kiel. Dr. Klaus Schrader leitet dort den Bereich Schwerpunktanalysen.


Literatur

BMF (Bundesministerium der Finanzen) (2020), Eckpunkte des Konjunkturprogramms: Corona-Folgen bekämpfen, Wohlstand sichern, Zukunftsfähigkeit stärken, https://www.bundesfinanzministerium.de/Content/DE/Standardartikel/Themen/Schlaglichter/Konjunkturpaket/2020-06-03-eckpunktepapier.html (7. Juli 2020).
Boysen-Hogrefe J Zum Fiskalimpuls des Konjunkturpakets Wirtschaftsdienst 2020 100 7 599 600 10.1007/s10273-020-2704-5
Felbermayr, G. (2020), Konjunkturpakete und Klimaschutz gehören nicht vermengt, Wirtschaftswoche online, 3. Juni, https://www.wiwo.de/politik/konjunktur/welt-wirtschaft-konjunkturpakete-und-klimaschutz-gehoeren-nicht-vermengt/25884246.html (7. Juli 2020).
Laaser, C.-F. und A. Rosenschon (2018), Kieler Subventionsbericht und die Kieler Subventionsampel: Finanzhilfen des Bundes und Steuervergünstigungen bis 2017 — eine Aktualisierung, Kieler Beiträge zur Wirtschaftspolitik, 14, https://www.ifw-kiel.de/fileadmin/Dateiverwaltung/IfW-Publications/-ifw/Kieler_Beitraege_zur_Wirtschaftspolitik/wipo_14.pdf (7. Juli 2020).
Laaser, C.-F. und A. Rosenschon (2019), Kieler Subventionsbericht: Steigende Subventionen des Bundes bis zum Jahr 2018, Mit einer Schwerpunktanalyse Verkehrssubventionen, Kieler Beiträge zur Wirtschaftspolitik, 22, https://www.ifw-kiel.de/fileadmin/Dateiverwaltung/IfW-Publications/-ifw/Kieler_Beitraege_zur_Wirtschaftspolitik/wipo_22.pdf (7. Juli 2020).
Laaser, C.-F. und A. Rosenschon (2020), Der Kieler Subventionsbericht 2020 — Ergänzungen nach dem Schweizer Modell, Kieler Beitrag zur Wirtschaftspolitik, im Erscheinen.
